# (4-Fluoro­phen­yl)thio­urea–1,10-phenanthroline (1/1)

**DOI:** 10.1107/S1600536813023222

**Published:** 2013-08-23

**Authors:** Bohari M. Yamin, Halima F. Salem, Siti Fairus M. Yusoff

**Affiliations:** a School of Chemical Sciences and Food Technology, Universiti Kebangsaan Malaysia, 43600 Bangi, Selangor, Malaysia

## Abstract

Refluxing a mixture of 1,10-phenanthroline, (4-fluoro­phen­yl)thio­urea and cadmium(II) chloride did not produce the expected mixed-ligand complex but formed a co-crystal of the two ligands, C_12_H_8_N_2_·C_7_H_7_FN_2_S. The asymmetric unit consists of two pairs of the co-crystal mol­ecules. In each (4-fluoro­phen­yl)thio­urea mol­ecule, the planes of the N_2_CS thio­urea units are almost perpendicular to the corresponding fluoro­benzene rings, subtending angles of 76.53 (7) and 85.25 (7)°. In the crystal, N—H⋯N and N—H⋯S hydrogen bonds form inversion dimers from the co-crystal pairs. A weak π–π inter­action between the phenanthroline rings [centroid–centroid distance = 3.7430 (15)Å] is also observed.

## Related literature

For bond-length data, see: Allen *et al.* (1987 ▶). For related structures of other co-crystals formed with 1,10-phenanthroline, see: Ton & Bolte (2005 ▶); Wang *et al.* (2006 ▶); Shan *et al.* (2001 ▶).
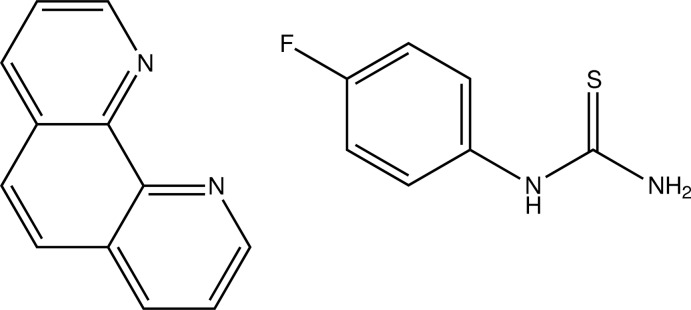


## Experimental

### 

#### Crystal data

C_12_H_8_N_2_·C_7_H_7_FN_2_S
*M* *_r_* = 350.41
Triclinic, 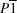

*a* = 10.245 (3) Å
*b* = 12.720 (3) Å
*c* = 15.222 (4) Å
α = 69.523 (4)°
β = 75.854 (5)°
γ = 66.565 (4)°
*V* = 1692.2 (8) Å^3^
*Z* = 4
Mo *K*α radiation
μ = 0.21 mm^−1^
*T* = 298 K
0.50 × 0.49 × 0.41 mm

#### Data collection

Bruker SMART APEX CCD area-detector diffractometer
Absorption correction: multi-scan (*SADABS*; Bruker 2000 ▶) *T*_min_ = 0.902, *T*_max_ = 0.919
20024 measured reflections
6992 independent reflections
5255 reflections with *I* > 2σ(*I*)
*R*_int_ = 0.022

#### Refinement

*R*[*F* ^2^ > 2σ(*F* ^2^)] = 0.041
*wR*(*F* ^2^) = 0.121
*S* = 1.03
6992 reflections
451 parameters
H-atom parameters constrained
Δρ_max_ = 0.23 e Å^−3^
Δρ_min_ = −0.22 e Å^−3^

### 

Data collection: *SMART* (Bruker, 2000 ▶); cell refinement: *SAINT* (Bruker, 2000 ▶); data reduction: *SAINT*; program(s) used to solve structure: *SHELXTL* (Sheldrick, 2008 ▶); program(s) used to refine structure: *SHELXTL*; molecular graphics: *SHELXTL*; software used to prepare material for publication: *SHELXTL*, *PARST* (Nardelli, 1995 ▶) and *PLATON* (Spek, 2009 ▶).

## Supplementary Material

Crystal structure: contains datablock(s) global, I. DOI: 10.1107/S1600536813023222/sj5347sup1.cif

Structure factors: contains datablock(s) I. DOI: 10.1107/S1600536813023222/sj5347Isup2.hkl

Supplementary material file. DOI: 10.1107/S1600536813023222/sj5347Isup3.cml

Additional supplementary materials:  crystallographic information; 3D view; checkCIF report

## Figures and Tables

**Table 1:** Hydrogen-bond geometry (Å, °)

*D*—H⋯*A*	*D*—H	H⋯*A*	*D*⋯*A*	*D*—H⋯*A*
N1—H1*A*⋯N5^i^	0.86	2.28	3.076 (2)	155
N2—H2*A*⋯N6^i^	0.86	2.08	2.870 (2)	152
N2—H2*B*⋯S1^ii^	0.86	2.67	3.4770 (19)	158
N3—H3⋯N8^iii^	0.86	2.36	3.186 (2)	161
N4—H4*A*⋯N7^iii^	0.86	2.11	2.889 (2)	150
N4—H4*B*⋯S2^iv^	0.86	2.67	3.485 (2)	157

Symmetry codes: (i) 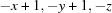; (ii) 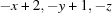; (iii) 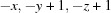; (iv) 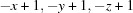.

**Fig. 1.**
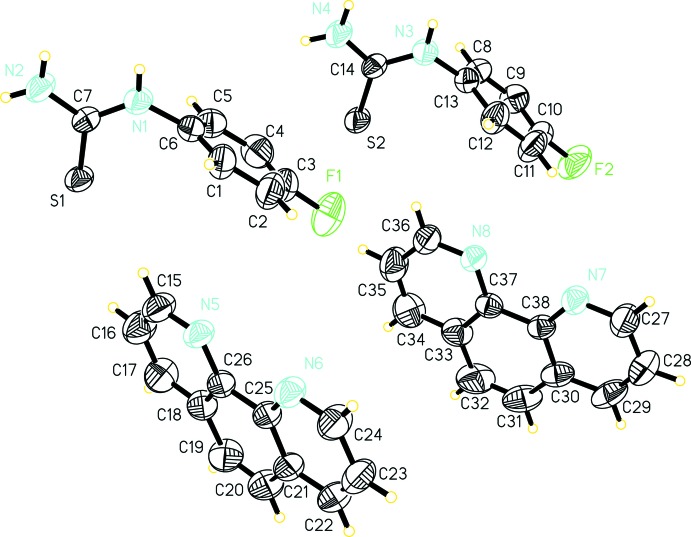
: Molecular structure of 1,10 phenanthroline-(4-fluorophenyl)thiourea co-crystal with 50% probability displacement ellipsoids

**Fig. 2.**
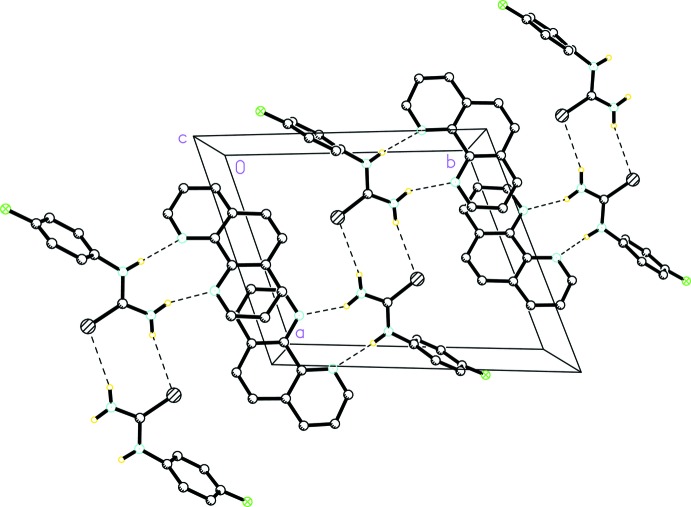
: Molecular packing of the 1, 10 phenanthroline-(4-fluorophenyl)thiourea co-crystal in the unit cell viewed along the c axis

## supplementary crystallographic information

### 1. Comment

1,10-phenantroline is a strong bidentate ligand capable of coordinating with many metals and subsequently promoting other ligands to complete the coordination sphere of a complex. On the other hand it can also form adducts or co-crystals with other compounds in the presence or absence of metal salts. Examples include 1,10-phenanthroline-chloroform (Ton & Bolte, 2005), 1,10-phenantroline-(2R,3R) tartaric acid trihydrate (Wang *et al.*, 2006) and nitrilotriacetic acid-1,10-phenantroline-H_2_O(1/1/1) (Shan *et al.*, 2001). The formation of co-crystals can also result in the display of an extensive network of hydrogen bonds.

The title compound is a co-crystal of 1,10-phenanthroline with 4-fluorophenylthiourea (Fig. 1) obtained from the reaction of 1,10-phenanthroline, p-fluorophenylthiourea and cadmium (II) chloride. There are two independent pairs of co-crystal molecules in the asymmetric unit. The phenanthroline molecules are planar with a maximum deviation of 0.040 (2)Å for atom C2 from the least squares plane of the ring system. The bond lengths are in normal ranges (Allen *et al.*, 1987). There is a weak C36-H36···F1 intramolecular hydrogen bond in one of the thiourea molecules.

In the crystal structure, the thiourea molecules are linked by intermolecular N–H···S hydrogen bonds and also to the phenanthrolines by N–H···N interactions (symmetry codes as shown in Table 2) forming inversion dimers (Fig. 2). In addition, there is a weak π–π stacking interaction between the phenanthroline ring centroids Cg2 (N6/C21-C25) and Cg3 (C18-C26) (symmetry code: 1-x,-y, 1-z) with a distance between the centroids of 3.7430 (15)Å.

### 2. Experimental

A mixture of (4-fluorophenyl)thiourea (2 mmol, 0.34 g) with cadmium (II) chloride (1 mmol, 0.34 g) in methanol was stirred for 1 h. 1,10-phenanthroline (1 mmol, 0.19 g) was then added to the mixture and refluxed for 3 hrs. The green precipitate was filtered and washed with cold ethanol. After recrystallization from ethanol colorless crystals were obtained after 3 days. Melting point: 457.1–458.3 K.

### 3. Refinement

After location in the difference map, the H-atoms attached to the C and N atoms were fixed geometrically at ideal positions and allowed to ride on the parent atoms with C—H = 0.93 Å, N—H = 0.86 Å and with *U*_iso_(H)=1.2*U*_eq_(C or N).

### Crystal data

**Table:**

C_12_H_8_N_2_·C_7_H_7_FN_2_S	*V* = 1692.2 (8) Å^3^
*M**_r_* = 350.41	*Z* = 4
Triclinic, *P*1	*F*(000) = 728
Hall symbol: -P 1	*D*_x_ = 1.375 Mg m^−^^3^
*a* = 10.245 (3) Å	Mo *K*α radiation, λ = 0.71073 Å
*b* = 12.720 (3) Å	µ = 0.21 mm^−^^1^
*c* = 15.222 (4) Å	*T* = 298 K
α = 69.523 (4)°	Block, colorless
β = 75.854 (5)°	0.50 × 0.49 × 0.41 mm
γ = 66.565 (4)°	

### Data collection

**Table:**

Bruker SMART APEX CCD area-detector diffractometer	6992 independent reflections
Radiation source: fine-focus sealed tube	5255 reflections with *I* > 2σ(*I*)
Graphite monochromator	*R*_int_ = 0.022
Detector resolution: 83.66 pixels mm^-1^	θ_max_ = 26.5°, θ_min_ = 1.4°
ω scans	*h* = −12→12
Absorption correction: multi-scan (*SADABS*; Bruker 2000)	*k* = −15→15
*T*_min_ = 0.902, *T*_max_ = 0.919	*l* = −19→19
20024 measured reflections	

### Refinement

**Table:**

Refinement on *F*^2^	Primary atom site location: structure-invariant direct methods
Least-squares matrix: full	Secondary atom site location: difference Fourier map
*R*[*F*^2^ > 2σ(*F*^2^)] = 0.041	Hydrogen site location: inferred from neighbouring sites
*wR*(*F*^2^) = 0.121	H-atom parameters constrained
*S* = 1.03	*w* = 1/[σ^2^(*F*_o_^2^) + (0.0656*P*)^2^ + 0.2053*P*] where *P* = (*F*_o_^2^ + 2*F*_c_^2^)/3
6992 reflections	(Δ/σ)_max_ < 0.001
451 parameters	Δρ_max_ = 0.23 e Å^−^^3^
0 restraints	Δρ_min_ = −0.22 e Å^−^^3^

### Special details

**Table:**

Geometry. All e.s.d.'s (except the e.s.d. in the dihedral angle between two l.s. planes) are estimated using the full covariance matrix. The cell e.s.d.'s are taken into account individually in the estimation of e.s.d.'s in distances, angles and torsion angles; correlations between e.s.d.'s in cell parameters are only used when they are defined by crystal symmetry. An approximate (isotropic) treatment of cell e.s.d.'s is used for estimating e.s.d.'s involving l.s. planes.
Refinement. Refinement of *F*^2^ against ALL reflections. The weighted *R*-factor *wR* and goodness of fit *S* are based on *F*^2^, conventional *R*-factors *R* are based on *F*, with *F* set to zero for negative *F*^2^. The threshold expression of *F*^2^ > σ(*F*^2^) is used only for calculating *R*-factors(gt) *etc*. and is not relevant to the choice of reflections for refinement. *R*-factors based on *F*^2^ are statistically about twice as large as those based on *F*, and *R*- factors based on ALL data will be even larger.

### Fractional atomic coordinates and isotropic or equivalent isotropic displacement parameters (Å^2^)

**Table:**

	*x*	*y*	*z*	*U*_iso_*/*U*_eq_	
S1	0.84390 (4)	0.40139 (3)	0.08829 (3)	0.04813 (13)	
S2	0.35529 (4)	0.39478 (3)	0.58513 (3)	0.05227 (13)	
F1	0.33200 (16)	0.30480 (13)	0.35367 (10)	0.1092 (5)	
F2	−0.11926 (15)	0.26323 (12)	0.87533 (9)	0.0944 (4)	
N1	0.60344 (13)	0.59163 (11)	0.07689 (9)	0.0490 (3)	
H1A	0.5575	0.6656	0.0513	0.059*	
N2	0.79779 (14)	0.62376 (11)	−0.02026 (10)	0.0546 (4)	
H2A	0.7436	0.6967	−0.0409	0.066*	
H2B	0.8868	0.6010	−0.0429	0.066*	
N3	0.10549 (13)	0.57248 (11)	0.58379 (9)	0.0486 (3)	
H3	0.0520	0.6447	0.5598	0.058*	
N4	0.28874 (15)	0.62014 (12)	0.48166 (10)	0.0594 (4)	
H4A	0.2294	0.6917	0.4637	0.071*	
H4B	0.3766	0.6026	0.4564	0.071*	
N5	0.54581 (14)	0.17515 (11)	0.06797 (10)	0.0517 (3)	
N6	0.31190 (14)	0.12042 (11)	0.06436 (10)	0.0536 (3)	
N7	−0.18619 (14)	0.12214 (12)	0.58764 (10)	0.0533 (3)	
N8	0.06097 (14)	0.17567 (11)	0.55104 (9)	0.0487 (3)	
C1	0.43416 (18)	0.48519 (16)	0.12639 (12)	0.0583 (4)	
H1	0.4166	0.5089	0.0640	0.070*	
C2	0.3670 (2)	0.41244 (19)	0.19549 (15)	0.0709 (5)	
H2	0.3049	0.3861	0.1804	0.085*	
C3	0.3938 (2)	0.38038 (17)	0.28597 (14)	0.0682 (5)	
C4	0.4789 (2)	0.42092 (17)	0.31253 (13)	0.0667 (5)	
H4	0.4908	0.4008	0.3757	0.080*	
C5	0.54713 (19)	0.49301 (15)	0.24264 (12)	0.0559 (4)	
H5	0.6064	0.5212	0.2587	0.067*	
C6	0.52715 (16)	0.52285 (13)	0.14942 (11)	0.0452 (3)	
C7	0.74405 (15)	0.54630 (12)	0.04661 (10)	0.0404 (3)	
C8	0.05680 (18)	0.47833 (14)	0.75159 (12)	0.0520 (4)	
H8	0.1044	0.5191	0.7646	0.062*	
C9	0.0014 (2)	0.40073 (16)	0.82506 (12)	0.0599 (4)	
H9	0.0105	0.3890	0.8874	0.072*	
C10	−0.0670 (2)	0.34187 (16)	0.80363 (13)	0.0610 (5)	
C11	−0.0854 (2)	0.35812 (19)	0.71378 (14)	0.0715 (6)	
H11	−0.1338	0.3174	0.7015	0.086*	
C12	−0.0304 (2)	0.43677 (17)	0.64110 (12)	0.0614 (5)	
H12	−0.0427	0.4499	0.5791	0.074*	
C13	0.04237 (15)	0.49591 (13)	0.65946 (11)	0.0443 (3)	
C14	0.24468 (16)	0.53690 (12)	0.54835 (10)	0.0421 (3)	
C15	0.65684 (19)	0.19861 (15)	0.07450 (14)	0.0616 (5)	
H15	0.6570	0.2763	0.0461	0.074*	
C16	0.7741 (2)	0.11518 (17)	0.12096 (15)	0.0691 (5)	
H16	0.8493	0.1369	0.1237	0.083*	
C17	0.7753 (2)	0.00132 (17)	0.16212 (14)	0.0656 (5)	
H17	0.8525	−0.0564	0.1931	0.079*	
C18	0.66056 (18)	−0.02913 (14)	0.15793 (12)	0.0532 (4)	
C19	0.6560 (2)	−0.14757 (15)	0.19988 (13)	0.0647 (5)	
H19	0.7315	−0.2068	0.2317	0.078*	
C20	0.5457 (2)	−0.17479 (14)	0.19423 (13)	0.0647 (5)	
H20	0.5465	−0.2529	0.2212	0.078*	
C21	0.42669 (18)	−0.08640 (14)	0.14760 (12)	0.0523 (4)	
C22	0.3075 (2)	−0.11079 (16)	0.14253 (14)	0.0655 (5)	
H22	0.3054	−0.1881	0.1686	0.079*	
C23	0.1948 (2)	−0.02219 (17)	0.09966 (15)	0.0705 (5)	
H23	0.1153	−0.0376	0.0956	0.085*	
C24	0.2019 (2)	0.09265 (16)	0.06190 (14)	0.0650 (5)	
H24	0.1244	0.1534	0.0332	0.078*	
C25	0.42480 (16)	0.03254 (13)	0.10611 (11)	0.0457 (3)	
C26	0.54590 (16)	0.06138 (13)	0.10972 (11)	0.0455 (3)	
C27	−0.3040 (2)	0.09576 (18)	0.60676 (15)	0.0698 (5)	
H27	−0.3888	0.1577	0.5898	0.084*	
C28	−0.3099 (2)	−0.0184 (2)	0.65059 (16)	0.0756 (6)	
H28	−0.3961	−0.0322	0.6629	0.091*	
C29	−0.1868 (3)	−0.10883 (18)	0.67474 (13)	0.0701 (5)	
H29	−0.1876	−0.1862	0.7041	0.084*	
C30	−0.0579 (2)	−0.08632 (14)	0.65567 (12)	0.0548 (4)	
C31	0.0750 (3)	−0.17715 (16)	0.68034 (15)	0.0755 (6)	
H31	0.0783	−0.2556	0.7093	0.091*	
C32	0.1951 (2)	−0.15183 (17)	0.66274 (17)	0.0812 (6)	
H32	0.2802	−0.2131	0.6799	0.097*	
C33	0.19570 (19)	−0.03244 (15)	0.61797 (13)	0.0595 (4)	
C34	0.3194 (2)	−0.00273 (19)	0.60049 (16)	0.0785 (6)	
H34	0.4066	−0.0622	0.6158	0.094*	
C35	0.3117 (2)	0.11256 (19)	0.56135 (16)	0.0742 (6)	
H35	0.3925	0.1338	0.5504	0.089*	
C36	0.18013 (19)	0.19821 (16)	0.53802 (13)	0.0588 (4)	
H36	0.1758	0.2772	0.5113	0.071*	
C37	0.06725 (16)	0.06030 (13)	0.59197 (10)	0.0441 (3)	
C38	−0.06280 (17)	0.03267 (13)	0.61146 (10)	0.0439 (3)	

### Atomic displacement parameters (Å^2^)

**Table:**

	*U*^11^	*U*^22^	*U*^33^	*U*^12^	*U*^13^	*U*^23^
S1	0.0463 (2)	0.0332 (2)	0.0577 (3)	−0.01352 (16)	−0.00820 (17)	−0.00332 (16)
S2	0.0454 (2)	0.0362 (2)	0.0608 (3)	−0.01320 (17)	−0.00714 (18)	0.00245 (17)
F1	0.1178 (11)	0.1091 (11)	0.0894 (9)	−0.0710 (9)	0.0284 (8)	−0.0053 (8)
F2	0.1220 (11)	0.0937 (9)	0.0774 (8)	−0.0751 (9)	0.0245 (7)	−0.0164 (7)
N1	0.0432 (7)	0.0349 (6)	0.0592 (8)	−0.0108 (5)	−0.0088 (6)	−0.0034 (6)
N2	0.0452 (7)	0.0365 (7)	0.0706 (9)	−0.0167 (6)	−0.0084 (6)	0.0021 (6)
N3	0.0430 (7)	0.0394 (7)	0.0535 (8)	−0.0115 (5)	−0.0084 (6)	−0.0031 (6)
N4	0.0499 (8)	0.0390 (7)	0.0708 (9)	−0.0167 (6)	−0.0055 (7)	0.0063 (6)
N5	0.0487 (7)	0.0351 (6)	0.0665 (9)	−0.0159 (6)	−0.0085 (6)	−0.0063 (6)
N6	0.0495 (8)	0.0401 (7)	0.0662 (9)	−0.0178 (6)	−0.0094 (6)	−0.0048 (6)
N7	0.0475 (7)	0.0446 (7)	0.0621 (9)	−0.0179 (6)	−0.0062 (6)	−0.0064 (6)
N8	0.0465 (7)	0.0419 (7)	0.0550 (8)	−0.0174 (6)	−0.0037 (6)	−0.0100 (6)
C1	0.0554 (10)	0.0748 (12)	0.0535 (10)	−0.0327 (9)	0.0033 (8)	−0.0233 (9)
C2	0.0649 (12)	0.0887 (14)	0.0745 (13)	−0.0467 (11)	0.0108 (10)	−0.0306 (11)
C3	0.0645 (11)	0.0626 (11)	0.0660 (12)	−0.0286 (9)	0.0165 (9)	−0.0134 (9)
C4	0.0734 (12)	0.0608 (11)	0.0497 (10)	−0.0156 (9)	−0.0031 (9)	−0.0084 (8)
C5	0.0583 (10)	0.0493 (9)	0.0584 (10)	−0.0150 (8)	−0.0134 (8)	−0.0133 (8)
C6	0.0418 (8)	0.0371 (7)	0.0507 (9)	−0.0108 (6)	−0.0023 (6)	−0.0111 (7)
C7	0.0435 (8)	0.0372 (7)	0.0436 (8)	−0.0177 (6)	−0.0109 (6)	−0.0068 (6)
C8	0.0574 (10)	0.0504 (9)	0.0554 (10)	−0.0246 (8)	−0.0102 (8)	−0.0144 (7)
C9	0.0714 (12)	0.0624 (11)	0.0469 (9)	−0.0280 (9)	−0.0053 (8)	−0.0124 (8)
C10	0.0667 (11)	0.0594 (10)	0.0590 (11)	−0.0352 (9)	0.0125 (8)	−0.0172 (8)
C11	0.0808 (13)	0.0911 (15)	0.0713 (13)	−0.0596 (12)	0.0110 (10)	−0.0356 (11)
C12	0.0675 (11)	0.0830 (13)	0.0513 (10)	−0.0435 (10)	0.0018 (8)	−0.0254 (9)
C13	0.0396 (8)	0.0428 (8)	0.0497 (9)	−0.0144 (6)	−0.0043 (6)	−0.0127 (7)
C14	0.0441 (8)	0.0382 (7)	0.0438 (8)	−0.0178 (6)	−0.0105 (6)	−0.0036 (6)
C15	0.0567 (10)	0.0427 (9)	0.0837 (13)	−0.0211 (8)	−0.0110 (9)	−0.0096 (8)
C16	0.0541 (10)	0.0623 (11)	0.0927 (15)	−0.0232 (9)	−0.0172 (10)	−0.0148 (10)
C17	0.0541 (10)	0.0579 (11)	0.0754 (13)	−0.0128 (8)	−0.0204 (9)	−0.0069 (9)
C18	0.0535 (9)	0.0403 (8)	0.0544 (10)	−0.0112 (7)	−0.0077 (7)	−0.0050 (7)
C19	0.0656 (11)	0.0396 (9)	0.0692 (12)	−0.0093 (8)	−0.0160 (9)	0.0028 (8)
C20	0.0793 (13)	0.0324 (8)	0.0674 (11)	−0.0178 (8)	−0.0097 (9)	0.0023 (8)
C21	0.0615 (10)	0.0381 (8)	0.0531 (9)	−0.0221 (7)	0.0001 (8)	−0.0071 (7)
C22	0.0754 (13)	0.0457 (9)	0.0756 (12)	−0.0330 (9)	0.0032 (10)	−0.0115 (9)
C23	0.0630 (11)	0.0633 (12)	0.0935 (15)	−0.0365 (10)	−0.0042 (10)	−0.0186 (10)
C24	0.0561 (10)	0.0542 (10)	0.0828 (13)	−0.0229 (8)	−0.0137 (9)	−0.0091 (9)
C25	0.0503 (9)	0.0336 (7)	0.0470 (8)	−0.0157 (6)	−0.0014 (7)	−0.0058 (6)
C26	0.0472 (8)	0.0346 (7)	0.0472 (8)	−0.0128 (6)	−0.0021 (6)	−0.0065 (6)
C27	0.0556 (11)	0.0637 (11)	0.0888 (14)	−0.0270 (9)	−0.0083 (10)	−0.0132 (10)
C28	0.0738 (13)	0.0792 (14)	0.0837 (14)	−0.0488 (12)	−0.0010 (11)	−0.0145 (11)
C29	0.1043 (16)	0.0578 (11)	0.0610 (11)	−0.0537 (12)	−0.0068 (10)	−0.0041 (9)
C30	0.0777 (12)	0.0406 (8)	0.0474 (9)	−0.0240 (8)	−0.0132 (8)	−0.0060 (7)
C31	0.1035 (17)	0.0368 (9)	0.0818 (14)	−0.0210 (10)	−0.0334 (12)	−0.0005 (9)
C32	0.0824 (15)	0.0434 (10)	0.1036 (17)	−0.0014 (10)	−0.0415 (13)	−0.0071 (10)
C33	0.0568 (10)	0.0493 (9)	0.0679 (11)	−0.0087 (8)	−0.0197 (9)	−0.0141 (8)
C34	0.0517 (11)	0.0726 (13)	0.1020 (17)	−0.0063 (10)	−0.0267 (11)	−0.0198 (12)
C35	0.0500 (10)	0.0824 (14)	0.0930 (15)	−0.0270 (10)	−0.0106 (10)	−0.0220 (12)
C36	0.0552 (10)	0.0557 (10)	0.0660 (11)	−0.0259 (8)	−0.0032 (8)	−0.0126 (8)
C37	0.0493 (8)	0.0387 (8)	0.0412 (8)	−0.0120 (6)	−0.0077 (6)	−0.0098 (6)
C38	0.0546 (9)	0.0380 (8)	0.0388 (8)	−0.0180 (7)	−0.0063 (6)	−0.0077 (6)

### Geometric parameters (Å, º)

**Table:**

S1—C7	1.6869 (15)	C12—C13	1.377 (2)
S2—C14	1.6847 (15)	C12—H12	0.9300
F1—C3	1.364 (2)	C15—C16	1.395 (3)
F2—C10	1.3620 (19)	C15—H15	0.9300
N1—C7	1.3494 (19)	C16—C17	1.358 (2)
N1—C6	1.4288 (19)	C16—H16	0.9300
N1—H1A	0.8601	C17—C18	1.397 (2)
N2—C7	1.3289 (18)	C17—H17	0.9300
N2—H2A	0.8601	C18—C26	1.414 (2)
N2—H2B	0.8601	C18—C19	1.431 (2)
N3—C14	1.3457 (19)	C19—C20	1.336 (3)
N3—C13	1.4303 (19)	C19—H19	0.9300
N3—H3	0.8600	C20—C21	1.424 (2)
N4—C14	1.3265 (18)	C20—H20	0.9300
N4—H4A	0.8600	C21—C22	1.399 (2)
N4—H4B	0.8599	C21—C25	1.415 (2)
N5—C15	1.319 (2)	C22—C23	1.359 (3)
N5—C26	1.3616 (18)	C22—H22	0.9300
N6—C24	1.320 (2)	C23—C24	1.395 (2)
N6—C25	1.351 (2)	C23—H23	0.9300
N7—C27	1.317 (2)	C24—H24	0.9300
N7—C38	1.347 (2)	C25—C26	1.444 (2)
N8—C36	1.316 (2)	C27—C28	1.389 (3)
N8—C37	1.3604 (19)	C27—H27	0.9300
C1—C6	1.380 (2)	C28—C29	1.351 (3)
C1—C2	1.381 (2)	C28—H28	0.9300
C1—H1	0.9300	C29—C30	1.402 (3)
C2—C3	1.357 (3)	C29—H29	0.9300
C2—H2	0.9300	C30—C38	1.410 (2)
C3—C4	1.366 (3)	C30—C31	1.423 (3)
C4—C5	1.387 (2)	C31—C32	1.335 (3)
C4—H4	0.9300	C31—H31	0.9300
C5—C6	1.379 (2)	C32—C33	1.433 (3)
C5—H5	0.9300	C32—H32	0.9300
C8—C13	1.376 (2)	C33—C34	1.402 (3)
C8—C9	1.382 (2)	C33—C37	1.405 (2)
C8—H8	0.9300	C34—C35	1.353 (3)
C9—C10	1.363 (2)	C34—H34	0.9300
C9—H9	0.9300	C35—C36	1.388 (3)
C10—C11	1.359 (3)	C35—H35	0.9300
C11—C12	1.383 (2)	C36—H36	0.9300
C11—H11	0.9300	C37—C38	1.447 (2)
			
C7—N1—C6	123.25 (12)	C16—C17—H17	120.0
C7—N1—H1A	118.4	C18—C17—H17	120.0
C6—N1—H1A	118.4	C17—C18—C26	118.00 (15)
C7—N2—H2A	120.0	C17—C18—C19	122.47 (16)
C7—N2—H2B	120.0	C26—C18—C19	119.53 (16)
H2A—N2—H2B	120.0	C20—C19—C18	121.38 (16)
C14—N3—C13	123.08 (12)	C20—C19—H19	119.3
C14—N3—H3	118.5	C18—C19—H19	119.3
C13—N3—H3	118.5	C19—C20—C21	121.21 (15)
C14—N4—H4A	120.0	C19—C20—H20	119.4
C14—N4—H4B	120.0	C21—C20—H20	119.4
H4A—N4—H4B	120.0	C22—C21—C25	117.46 (16)
C15—N5—C26	117.52 (14)	C22—C21—C20	122.83 (15)
C24—N6—C25	118.23 (14)	C25—C21—C20	119.69 (16)
C27—N7—C38	118.10 (15)	C23—C22—C21	120.41 (16)
C36—N8—C37	117.39 (14)	C23—C22—H22	119.8
C6—C1—C2	120.36 (17)	C21—C22—H22	119.8
C6—C1—H1	119.8	C22—C23—C24	117.96 (17)
C2—C1—H1	119.8	C22—C23—H23	121.0
C3—C2—C1	118.37 (18)	C24—C23—H23	121.0
C3—C2—H2	120.8	N6—C24—C23	124.09 (17)
C1—C2—H2	120.8	N6—C24—H24	118.0
C2—C3—F1	118.43 (19)	C23—C24—H24	118.0
C2—C3—C4	123.11 (17)	N6—C25—C21	121.84 (15)
F1—C3—C4	118.46 (19)	N6—C25—C26	118.98 (13)
C3—C4—C5	118.14 (18)	C21—C25—C26	119.18 (14)
C3—C4—H4	120.9	N5—C26—C18	121.72 (15)
C5—C4—H4	120.9	N5—C26—C25	119.30 (13)
C6—C5—C4	120.10 (17)	C18—C26—C25	118.97 (14)
C6—C5—H5	120.0	N7—C27—C28	124.33 (19)
C4—C5—H5	120.0	N7—C27—H27	117.8
C5—C6—C1	119.77 (15)	C28—C27—H27	117.8
C5—C6—N1	120.11 (15)	C29—C28—C27	118.08 (18)
C1—C6—N1	120.12 (15)	C29—C28—H28	121.0
N2—C7—N1	115.02 (13)	C27—C28—H28	121.0
N2—C7—S1	122.23 (12)	C28—C29—C30	120.17 (17)
N1—C7—S1	122.73 (11)	C28—C29—H29	119.9
C13—C8—C9	120.71 (15)	C30—C29—H29	119.9
C13—C8—H8	119.6	C29—C30—C38	117.62 (17)
C9—C8—H8	119.6	C29—C30—C31	122.98 (17)
C10—C9—C8	118.18 (16)	C38—C30—C31	119.39 (17)
C10—C9—H9	120.9	C32—C31—C30	121.25 (17)
C8—C9—H9	120.9	C32—C31—H31	119.4
C11—C10—F2	118.42 (17)	C30—C31—H31	119.4
C11—C10—C9	122.92 (16)	C31—C32—C33	121.49 (18)
F2—C10—C9	118.66 (17)	C31—C32—H32	119.3
C10—C11—C12	118.23 (16)	C33—C32—H32	119.3
C10—C11—H11	120.9	C34—C33—C37	117.82 (16)
C12—C11—H11	120.9	C34—C33—C32	122.76 (17)
C13—C12—C11	120.72 (17)	C37—C33—C32	119.40 (17)
C13—C12—H12	119.6	C35—C34—C33	119.90 (17)
C11—C12—H12	119.6	C35—C34—H34	120.1
C8—C13—C12	119.21 (15)	C33—C34—H34	120.1
C8—C13—N3	120.19 (14)	C34—C35—C36	118.24 (18)
C12—C13—N3	120.58 (14)	C34—C35—H35	120.9
N4—C14—N3	115.87 (13)	C36—C35—H35	120.9
N4—C14—S2	122.12 (12)	N8—C36—C35	124.70 (17)
N3—C14—S2	121.99 (11)	N8—C36—H36	117.6
N5—C15—C16	124.64 (16)	C35—C36—H36	117.6
N5—C15—H15	117.7	N8—C37—C33	121.92 (15)
C16—C15—H15	117.7	N8—C37—C38	119.07 (13)
C17—C16—C15	118.11 (17)	C33—C37—C38	118.99 (14)
C17—C16—H16	120.9	N7—C38—C30	121.70 (15)
C15—C16—H16	120.9	N7—C38—C37	118.82 (13)
C16—C17—C18	120.01 (16)	C30—C38—C37	119.48 (14)
			
C6—C1—C2—C3	0.6 (3)	C22—C21—C25—N6	1.1 (2)
C1—C2—C3—F1	−177.47 (17)	C20—C21—C25—N6	−177.38 (16)
C1—C2—C3—C4	3.0 (3)	C22—C21—C25—C26	−179.92 (15)
C2—C3—C4—C5	−3.6 (3)	C20—C21—C25—C26	1.6 (2)
F1—C3—C4—C5	176.85 (16)	C15—N5—C26—C18	−0.4 (2)
C3—C4—C5—C6	0.6 (3)	C15—N5—C26—C25	178.58 (15)
C4—C5—C6—C1	2.9 (2)	C17—C18—C26—N5	0.2 (2)
C4—C5—C6—N1	−176.58 (15)	C19—C18—C26—N5	−179.44 (16)
C2—C1—C6—C5	−3.5 (3)	C17—C18—C26—C25	−178.77 (16)
C2—C1—C6—N1	175.93 (16)	C19—C18—C26—C25	1.6 (2)
C7—N1—C6—C5	74.5 (2)	N6—C25—C26—N5	−2.4 (2)
C7—N1—C6—C1	−104.90 (18)	C21—C25—C26—N5	178.51 (14)
C6—N1—C7—N2	−178.91 (14)	N6—C25—C26—C18	176.56 (14)
C6—N1—C7—S1	2.5 (2)	C21—C25—C26—C18	−2.5 (2)
C13—C8—C9—C10	0.4 (3)	C38—N7—C27—C28	0.4 (3)
C8—C9—C10—C11	−1.4 (3)	N7—C27—C28—C29	−0.5 (3)
C8—C9—C10—F2	178.58 (16)	C27—C28—C29—C30	0.1 (3)
F2—C10—C11—C12	−179.10 (17)	C28—C29—C30—C38	0.3 (3)
C9—C10—C11—C12	0.9 (3)	C28—C29—C30—C31	179.2 (2)
C10—C11—C12—C13	0.7 (3)	C29—C30—C31—C32	−178.6 (2)
C9—C8—C13—C12	1.1 (2)	C38—C30—C31—C32	0.2 (3)
C9—C8—C13—N3	−177.32 (15)	C30—C31—C32—C33	−0.3 (3)
C11—C12—C13—C8	−1.7 (3)	C31—C32—C33—C34	178.5 (2)
C11—C12—C13—N3	176.77 (16)	C31—C32—C33—C37	0.0 (3)
C14—N3—C13—C8	81.4 (2)	C37—C33—C34—C35	1.2 (3)
C14—N3—C13—C12	−97.02 (19)	C32—C33—C34—C35	−177.4 (2)
C13—N3—C14—N4	−176.57 (14)	C33—C34—C35—C36	−1.2 (3)
C13—N3—C14—S2	4.5 (2)	C37—N8—C36—C35	1.3 (3)
C26—N5—C15—C16	0.1 (3)	C34—C35—C36—N8	−0.1 (3)
N5—C15—C16—C17	0.4 (3)	C36—N8—C37—C33	−1.2 (2)
C15—C16—C17—C18	−0.6 (3)	C36—N8—C37—C38	177.18 (14)
C16—C17—C18—C26	0.3 (3)	C34—C33—C37—N8	0.0 (3)
C16—C17—C18—C19	179.92 (18)	C32—C33—C37—N8	178.62 (16)
C17—C18—C19—C20	−179.41 (19)	C34—C33—C37—C38	−178.38 (16)
C26—C18—C19—C20	0.2 (3)	C32—C33—C37—C38	0.2 (3)
C18—C19—C20—C21	−1.1 (3)	C27—N7—C38—C30	0.0 (2)
C19—C20—C21—C22	−178.19 (18)	C27—N7—C38—C37	−179.29 (16)
C19—C20—C21—C25	0.2 (3)	C29—C30—C38—N7	−0.3 (2)
C25—C21—C22—C23	−0.5 (3)	C31—C30—C38—N7	−179.25 (16)
C20—C21—C22—C23	177.94 (18)	C29—C30—C38—C37	178.92 (15)
C21—C22—C23—C24	−0.4 (3)	C31—C30—C38—C37	0.0 (2)
C25—N6—C24—C23	−0.1 (3)	N8—C37—C38—N7	0.6 (2)
C22—C23—C24—N6	0.7 (3)	C33—C37—C38—N7	179.06 (15)
C24—N6—C25—C21	−0.8 (2)	N8—C37—C38—C30	−178.69 (13)
C24—N6—C25—C26	−179.80 (16)	C33—C37—C38—C30	−0.2 (2)

### Hydrogen-bond geometry (Å, º)

**Table:**

*D*—H···*A*	*D*—H	H···*A*	*D*···*A*	*D*—H···*A*
C36—H36···F1	0.93	2.53	3.003 (3)	112
N1—H1*A*···N5^i^	0.86	2.28	3.076 (2)	155
N2—H2*A*···N6^i^	0.86	2.08	2.870 (2)	152
N2—H2*B*···S1^ii^	0.86	2.67	3.4770 (19)	158
N3—H3···N8^iii^	0.86	2.36	3.186 (2)	161
N4—H4*A*···N7^iii^	0.86	2.11	2.889 (2)	150
N4—H4*B*···S2^iv^	0.86	2.67	3.485 (2)	157

Symmetry codes: (i) −*x*+1, −*y*+1, −*z*; (ii) −*x*+2, −*y*+1, −*z*; (iii) −*x*, −*y*+1, −*z*+1; (iv) −*x*+1, −*y*+1, −*z*+1.
